# Quercetin-Induced Cell Death in Human Papillary Thyroid Cancer (B-CPAP) Cells

**DOI:** 10.1155/2016/9843675

**Published:** 2016-01-20

**Authors:** Ergül Mutlu Altundağ, Tolga Kasacı, Ayşe Mine Yılmaz, Betül Karademir, Semra Koçtürk, Yavuz Taga, A. Süha Yalçın

**Affiliations:** ^1^Department of Biochemistry, School of Medicine, Marmara University, Maltepe, 34854 Istanbul, Turkey; ^2^Genetic and Metabolic Diseases Research Center, Marmara University, Maltepe, 34854 Istanbul, Turkey; ^3^Department of Biochemistry, School of Medicine, Dokuz Eylül University, Inciralti, 35340 Izmir, Turkey

## Abstract

In this study, we have investigated the antiproliferative effect of quercetin on human papillary thyroid cancer cells and determined the apoptotic mechanisms underlying its actions. We have used different concentrations of quercetin to induce apoptosis and measured cell viability. Apoptosis and cell cycle analysis was determined by flow cytometry using Annexin V and propidium iodide. Finally, we have measured changes in caspase-3 and cleaved poly(ADP-ribose) polymerase (PARP) protein expression levels as hallmarks of apoptosis and Hsp90 protein expression level as a marker of proteasome activity in treated and control cells. Quercetin treatment of human papillary thyroid cancer cells resulted in decreased cell proliferation and increased rate of apoptosis by caspase activation. Furthermore, it was demonstrated that quercetin induces cancer cell apoptosis by downregulating the levels of Hsp90. In conclusion, we have shown that quercetin induces downregulation of Hsp90 expression that may be involved in the decrease of chymotrypsin-like proteasome activity which, in order, induces inhibition of growth and causes cell death in thyroid cancer cells. Thus, quercetin appears to be a promising candidate drug for Hsp90 downregulation and apoptosis of thyroid cancer cells.

## 1. Introduction

Thyroid cancer represents 1% of all malignancies with papillary thyroid carcinoma forming the majority of cases [1]. B-CPAP is a papillary thyroid carcinoma cell line that is known to have BRAF V600E mutation. BRAF mutations occur in approximately 8% of human tumors and are also widespread in papillary thyroid cancer (36–69%) [2, 3]. Cancer therapy aimed at MAPK signaling utilizes selective inhibitors of RAF and MEK kinases as well as inhibitors of the Hsp90 [4–6]. Hsp90 is a chaperone protein necessary for survival under stress conditions which also regulates the stability and activity of oncogenic proteins [5]. It has been reported that inhibitors of Hsp90 may stimulate proteasomal degradation of mutant B-Raf proteins [6].

Protein degradation is an important aspect of chemotherapy since cancer cells may develop resistance via this pathway. Cancer cells have high proteasome activity compared to normal cells and the cancer cell degrades damaged proteins with its proteasome activity to develop resistance against chemotherapeutic drugs [5–7]. Many tumors show increased level of Hsps (Hsp90, Hsp70, Hsp60, and Hsp27) that promote tumor cell survival, growth, and metastasis [7, 8]. These proteins are also involved in the oncogenic pathways of thyroid cancers. Since Hsp90 is known to interact with the proteasomal degradation system, it becomes important to consider its chemotherapeutic role. Thus Hsp90 inhibition may provide a new approach to thyroid cancer treatment.

On the other hand, studies in animals and humans have suggested that polyphenols, in particular the flavonoids, play an important role in regulating tumorigenesis [7, 9]. Quercetin is a component of most eatable fruits and vegetables. It is a naturally occurring flavone that is present at high concentrations in different berries, onions, apples, and red wine [10]. Quercetin has selective antiproliferative and antitumor effects via apoptotic mechanisms on different human cancer cell lines [11]. In this study, we have used a papillary thyroid carcinoma cell line (B-CPAP) and investigated the antiproliferative effect of quercetin on these cells. We have determined the underlying apoptotic mechanisms which included caspase-3 activation, PARP cleavage, Hsp90 inhibition, and proteasomal degradation.

## 2. Materials and Methods

Propidium iodide (PI), quercetin (Q4951, purity > 95%), MCA (methylcoumarin), suc-LLVY-MCA (succinyl-leucine-leucine-valine-tyrosine-methylcoumarin), and ATP were purchased from Sigma-Aldrich (USA). WST-1 kit was from Roche Diagnostics (USA) and Hoechst 33342 stain from Life Technologies (Germany). Annexin V/PI was supplied by Millipore (USA) and formulated according to the manufacturer's instructions. Antibodies used were caspase-3 (Cat. #9662, Cell Signaling, USA), cleaved PARP (Cat. #9541, Cell Signaling, USA), Hsp90 (Cat. #4874, Cell Signaling, USA), *β*-actin (Cat. #4967, Cell Signaling, USA), and HRP-linked anti-rabbit IgG (Cat. #7074, Cell Signaling, USA). Human papillary thyroid cancer cells (B-CPAP) were obtained from Deutsche Sammlung von Mikroorganismen und Zellkulturen (DSMZ, ACC 273).

### 2.1. Cell Viability Assay

Cells were cultured in RPMI-1640 medium (Biochrom, Germany) supplemented with 10% fetal bovine serum (Hyclone Laboratories, USA), 1% L-glutamine, and 1% penicillin-streptomycin in a 5% CO_2_ incubator at 37°C. Cell viability was monitored using the WST-1 kit. Quercetin stock solution (500 mM) was prepared in dimethyl sulfoxide (DMSO) and stored at −20°C. It was diluted with RPMI-1640 medium before being used in different assays and the final concentration of DMSO was kept below 0.1%. In viability experiments, 7 × 10^3^ cells/well were seeded in 96-well plates in 100 *μ*L of culture media and were exposed to different concentrations of quercetin (10–200 *μ*M). After 24, 48, or 72 hours, 10 *μ*L of WST-1 was added to each well and incubated for 2 hours at 37°C. The plates were then shaken thoroughly on the shaker for 1 min and the absorbance of reporter substrate was measured at 420–480 nm using a microplate reader (Molecular Devices, USA).

### 2.2. Detection of Apoptosis, Cell Cycle Analysis, and Chromatin Staining

For detection of apoptosis, 0.5 × 10^6^ cells were washed with PBS and ApopNexin FITC apoptosis detection kit (Millipore, USA) was used for analysis. In each assay 1 × 10^4^ cells were measured. All experiments were performed in triplicate and results were assessed using the CellQuest program (Becton Dickinson, USA). For cell cycle analysis, 0.5 × 10^6^ cells were harvested and centrifuged and the supernatant was discarded [12]. The pellet was suspended in PBS and 70% cold ethanol. Cells were washed once with PBS, followed by incubation in PBS containing 50 mg/mL PI and 2 mg/mL DNase-free RNase A for 30 min at room temperature in the dark and they were analyzed on FACSCalibur flow cytometry system (Becton Dickinson, USA). For chromatin staining, cells were treated with quercetin for 24 hours and collected by centrifugation at 300 ×g for 5 min, fixed with 3.7% paraformaldehyde for 20 min, and then stained with 10 *μ*M Hoechst 33342 dye for 15 min. After washing with PBS, fluorescence was evaluated using a fluorescence microscope (Leica DFC 310 FX, Germany).

### 2.3. Western Blot Experiments and Proteasome Activity Measurements

For Western blotting and proteasome activity measurements, 1.8 × 10^6^ cells were plated on 10 cm culture dishes. Cells were harvested and lysed in 200 *μ*L cold lysis buffer (50 mM Tris–HCl, pH 6.8, 15 mM EDTA, 15 mM MgCl_2_, 50 mM *β*-glycerol, 150 *μ*g/mL digitonin containing 1 mM dithiothreitol, and 100 mM phenylmethylsulfonyl fluoride). Samples were incubated on ice for 15 min and the supernatant was collected after centrifugation at 18,000 ×g for 10 min. Protein concentration was determined using BCA assay (Pierce Chemical, USA). Approximately 30 *μ*g of total proteins was loaded to each well and SDS-PAGE analysis was performed according to Laemmli [13]. Proteins were then transferred to nitrocellulose membranes by Turbo-Blot system (Bio-Rad Laboratories, USA). Membranes were blocked with 5% skimmed milk in Tris-buffered saline containing 0.1% Tween 20 and immunoblotted overnight at 4°C with the primary antibodies (Hsp90, caspase-3, and cleaved PARP) followed by the appropriate horseradish peroxidase-linked secondary antibody. Detection was performed using the West Pico chemiluminescent substrate kit (Thermo Scientific, USA) and the ChemiDoc MP System (Bio-Rad Laboratories, USA).

For proteasome activity, cells were incubated in cold 225 mM Tris buffer (pH 7.8) containing 7.5 mM MgOAc, 7.5 mM MgCl_2_, 45 mM KCl, and 1 mM dithiothreitol, treated for 15 sec in liquid nitrogen and then for 1 min in 40°C water bath (3 times). The lysates were centrifuged at 15,000 ×g for 30 min at 4°C. The fluorogenic peptide succinyl-leucine-leucine-valine-tyrosine-methylcoumarin (suc-LLVY-MCA) was used as substrate at a concentration of 200 *μ*M to measure the chymotrypsin-like (CT-L) activity of the proteasome. To measure the activities of 20S and 26S proteasome, ATP was added into the reaction mixture. After 60 min of incubation at 37°C, methylcoumarin liberation was measured with a fluorescence reader at 360 nm excitation and 485 nm emission. Results were calculated using free methylcoumarin (MCA) as standard [14].

### 2.4. Statistical Analysis

The significance of the effects on treatment groups was compared by analysis of variance (ANOVA) followed by Tukey's multiple comparison and Bonferroni posttests. *p* values less than 0.05 were considered statistically significant.

## 3. Results

### 3.1. Effect of Quercetin on Cell Viability

**Figure 1:**
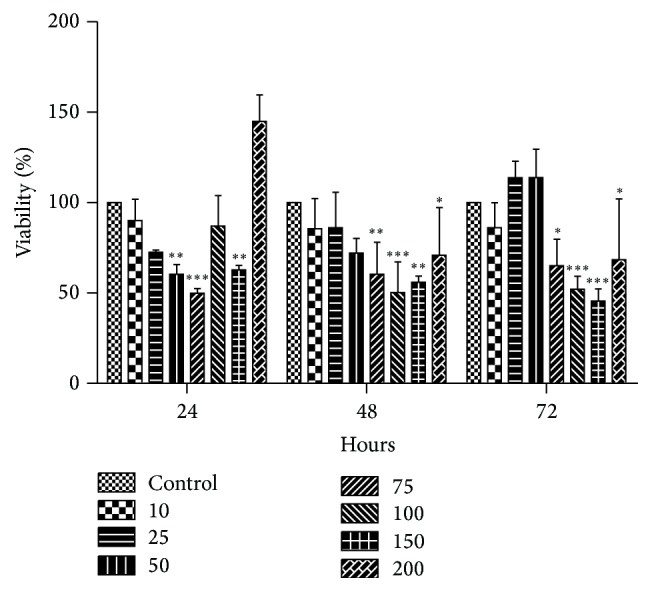
Effects of different concentrations of quercetin on cell viability. Cells were treated with different concentrations of quercetin (10–200 *μ*M) for 24, 48, and 72 hours and cell viability was measured by WST-1 assay. All data points represent mean ± SD, *n* = 3. Differences between means and significance of the treatments were analyzed using ANOVA and Bonferroni posttest. ^*∗*^ *p* < 0.05 versus control, ^*∗∗*^ *p* < 0.01 versus control, and ^*∗∗∗*^ *p* < 0.001 versus control.

Papillary thyroid cancer cells (B-CPAP) were treated with different concentrations of quercetin (10, 25, 50, 75, 100, 150, and 200 *μ*M) for 24, 48, and 72 hours to investigate the effect of quercetin on cell viability. As shown in Figure 1, quercetin treatment exerted both inhibitory and proliferative effects at different doses and time periods. A biphasic effect was observed after 24 hours. Although cell viability was decreased at 10, 25, 50, and 75 *μ*M concentrations compared to the control group, there was a significant proliferative effect at higher doses. These changes were less pronounced after 48 and 72 hours and were possibly masked by the proliferative effect and/or relatively short half-life of quercetin. In these preliminary experiments doubling time was observed to be 24 hours. Therefore, for further experiments, this time period and 10–75 *μ*M quercetin concentrations were chosen.

### 3.2. Apoptosis, Cell Cycle Analysis, and Chromatin Staining

**Figure 2:**
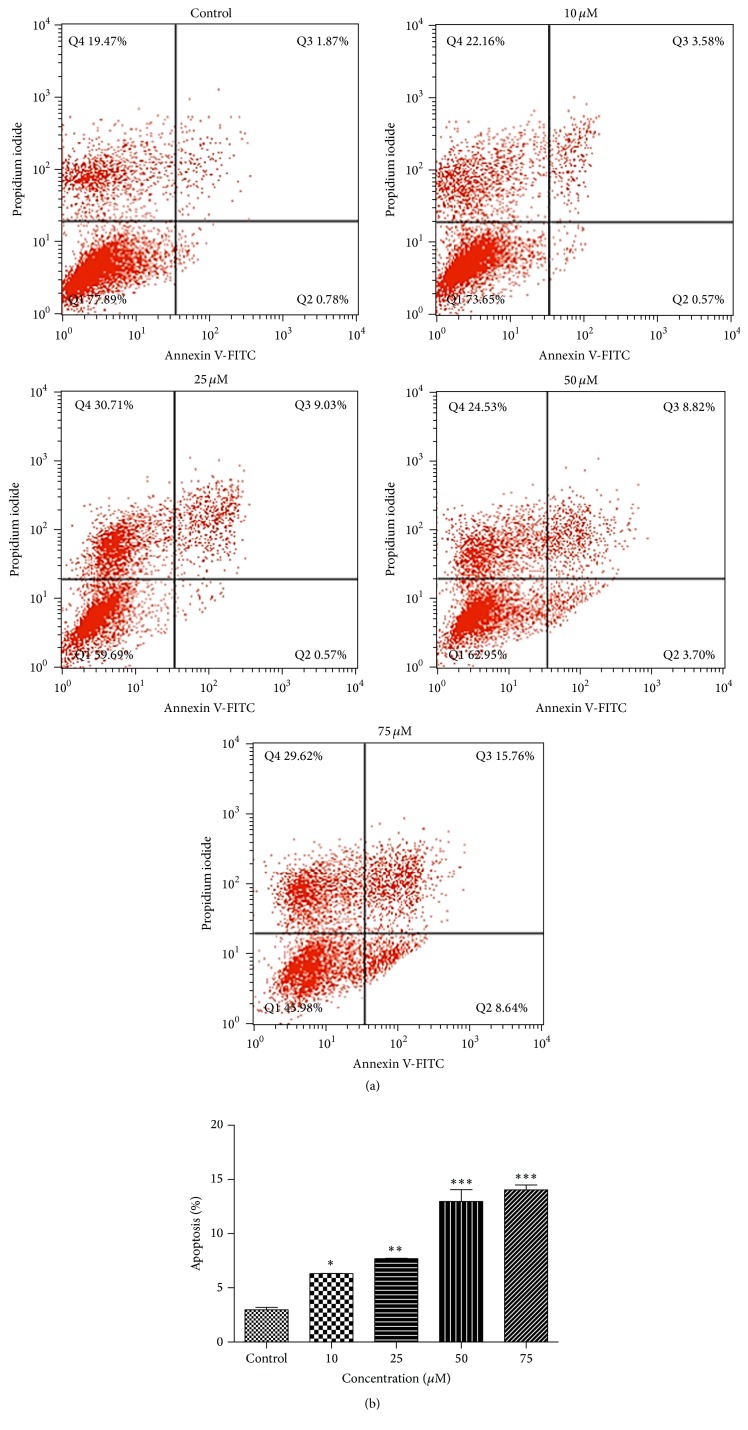
Flow cytometric analysis of quercetin-induced apoptotic cell death in B-CPAP cells. Cells were treated with different concentrations of quercetin (10–75 *μ*M) for 24 h. Apoptosis was evaluated using Annexin V-FITC and PI staining followed by flow cytometry (a). Cells in the lower left quadrant (Annexin V-FITC−/PI−) are viable; those in the lower right quadrant (Annexin V-FITC+/PI−) are early apoptotic and those in the upper right quadrants (Annexin V-FITC+/PI+) are late apoptotic or necrotic. Apoptosis bar graphs (b) represent mean ± SD, *n* = 3. Differences between means and significance of the treatments were analyzed using ANOVA and Tukey's multiple comparison test. ^*∗*^ *p* < 0.05 versus control, ^*∗∗*^ *p* < 0.01 versus control, and ^*∗∗∗*^ *p* < 0.001 versus control.

**Figure 3:**
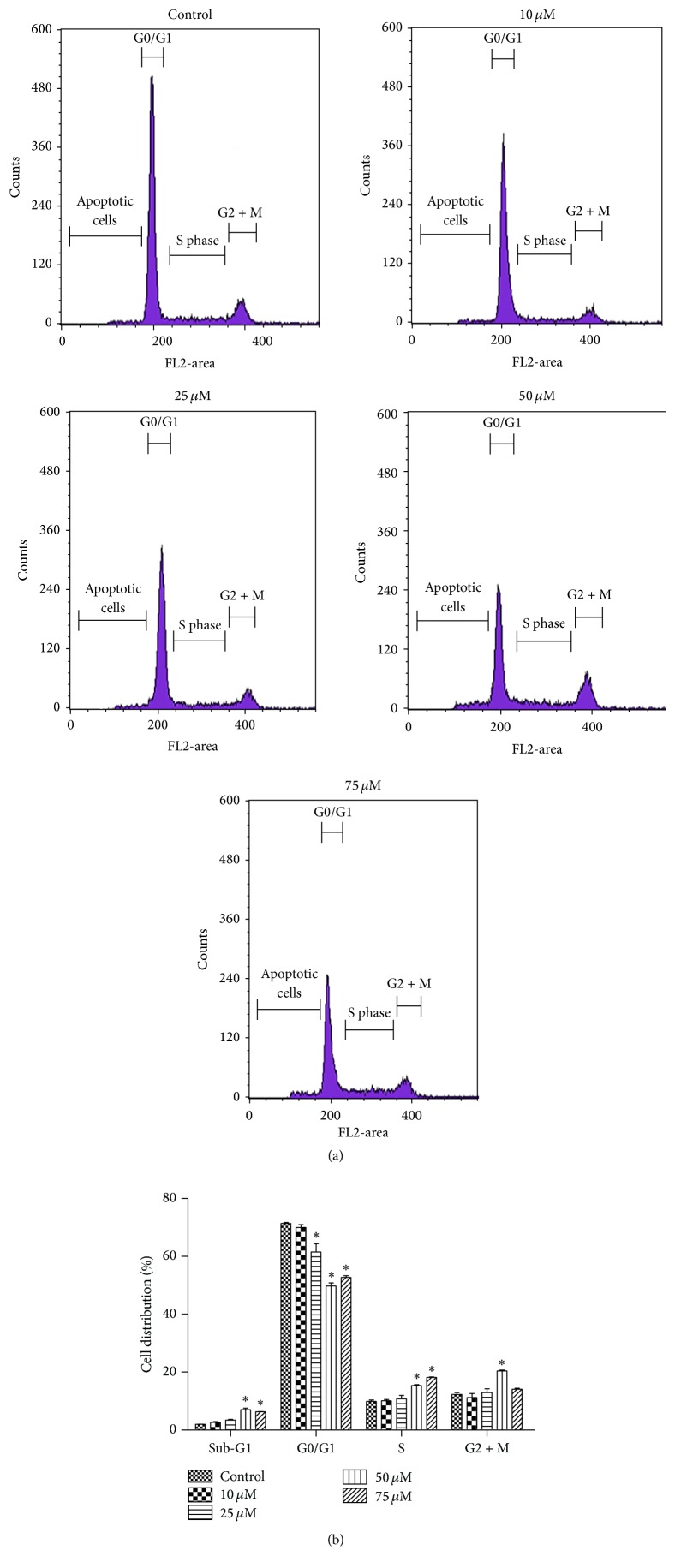
Flow cytometric analysis of quercetin-induced cell cycle distribution in B-CPAP cells. Cells were treated with different concentrations of quercetin (10–75 *μ*M) for 24 hours. Disruption of the cell cycle was analyzed by flow cytometry showing sub-G1 (apoptotic cells), G0/G1, S, and G2+M phases (a). Cell cycle bar graphs represent the percentage of cells within the different cell cycle phases (b). Differences between means and significance of the treatments were analyzed using ANOVA and Bonferroni posttest. Cell cycle bar graphs (b) represent mean ± SD, *n* = 3. ^*∗*^ *p* < 0.05 versus control.

**Figure 4:**
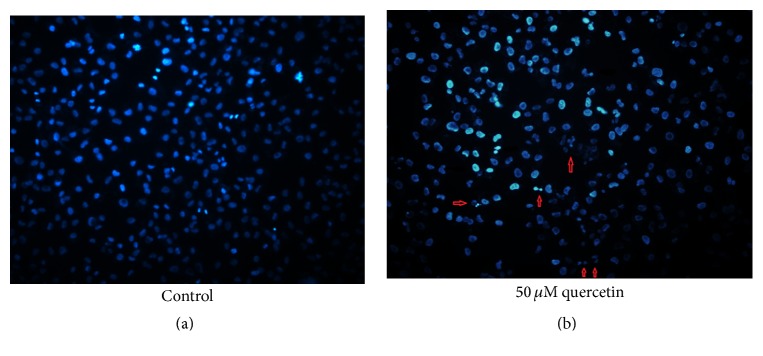
Chromatin staining of B-CPAP cells with Hoechst 33342. Arrows indicate blebbing and chromatin condensation. Fluorescence microscope: Leica DFC 310 FX, Magnification: 40x.

Figure 2 shows changes in percentage of cells undergoing apoptosis 24 hours after quercetin treatment at 10, 25, 50, and 75 *μ*M compared to the control group. B-CPAP cells stained with Annexin-V and PI were followed by flow cytometry to determine the percentage of apoptotic cell death. The percentage of early (Q2) and late (Q3) apoptotic cells illustrated quercetin-induced increase in apoptosis. Our results showed that percentage of cells undergoing apoptosis was increased at all quercetin concentrations compared to the control group, but apoptosis rates were high and similar at 50 and 75 *μ*M concentrations (Figure 2(b)). The results of cell cycle analysis showed that percentage distribution of cells in different phases was not changed at low concentrations of quercetin, but at 50 and 75 *μ*M concentrations the number of cells in sub-G1 and S phase was increased, whereas the number of G0/G1 phase cells was decreased (Figure 3). Chromatin staining with Hoechst 33342 showed morphological changes such as cell surface blebs and formation of apoptotic bodies after treatment of cells with 50 *μ*M quercetin (Figure 4).

### 3.3. Western Blot Analysis

**Figure 5:**
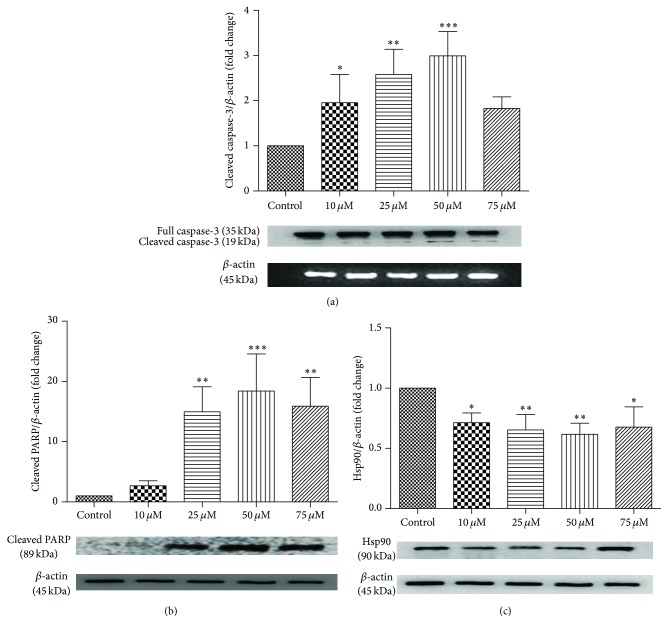
Western blot analysis of caspase-3 cleavage (a), PARP cleavage (b), and Hsp90 (c) protein expression levels after quercetin treatment of B-CPAP cells. Band intensity was analyzed by densitometry. Fold change of protein expression levels was calculated after bands were normalized to *β*-Actin. Differences between means and significance of the treatments were analyzed using ANOVA and Bonferroni posttest. Bar graphs represent mean ± SD, *n* = 3. ^*∗*^ *p* < 0.05 versus control, ^*∗∗*^ *p* < 0.01 versus control, and ^*∗∗∗*^ *p* < 0.001 versus control.

Figure 5 shows the results of Western blot analysis. Cleavage of caspase-3 and PARP confirmed apoptosis at higher concentrations. When the cells were exposed to 50 and 75 *μ*M quercetin, caspase-3 was cleaved from 35 to 19 kDa. Quercetin treatment downregulated the expression of Hsp90 in B-CPAP cells compared to controls. These results indicated that quercetin-induced cancer cell apoptosis may be related to the downregulation of Hsp90 levels.

### 3.4. Analysis of Proteasome Activity

**Figure 6:**
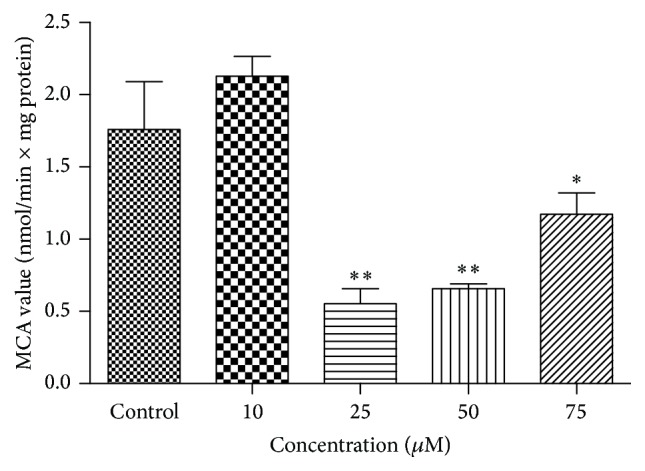
Determination of proteasome activity after quercetin treatment of B-CPAP cells. The fluorogenic peptide suc-LLVY-MCA was used as substrate to measure chymotrypsin-like (CT-L) activities of 20S and 26S proteasome. Differences between means and significance of the treatments were analyzed using ANOVA and Bonferroni posttest. Bar graphs represent mean ± SD, *n* = 3. ^*∗*^ *p* < 0.05 versus control, ^*∗∗*^ *p* < 0.01 versus control.

We have also measured the chymotrypsin-like proteasome activity (CT-L) of 20S and 26S proteasome in the presence of quercetin (Figure 6). CT-L was inhibited significantly at 25–75 *μ*M quercetin. Inhibition of Hsp90 by quercetin seems to be involved in the reduction of proteasome activity and apoptosis of B-CPAP cells.

## 4. Discussion

Differentiated thyroid carcinoma is the most common human endocrine malignancy of which papillary and follicular thyroid carcinomas are the two major variants [1]. Papillary thyroid cancers account for 80% of all thyroid cancers and have been characterized by alterations of one of several protein kinases [15]. Yin et al. [9] reported that flavonoids have potent antiproliferative activity* in vitro* against various human thyroid cancer cell lines and suggested that they might be used as therapeutic agents in the management of thyroid cancers. Although different groups studied apoptosis induced by quercetin in different cancer cell lines, the cellular and molecular mechanisms involved have not been fully elucidated [16, 17].

In this study we report antiproliferative effect of quercetin on human papillary thyroid cancer cells. Our results showed that quercetin inhibits proliferation especially at 50 and 75 *μ*M concentrations. Quercetin concentration that significantly decreased papillary thyroid cancer cell viability was similar to those observed on other cancer cells, such as hepatoma [16], lung [17], colon [18], leukemia, and breast [19]. In a previous study, it was shown that quercetin inhibits growth of different thyroid cancer cell lines [20]. Additionally, quercetin was reported as a potent polyphenol inducing apoptosis in leukemia cell lines with IC50 values ranging between 8 and 33 *μ*M and AP50 values (the concentration at which 50% of cells undergo apoptosis) ranging between 19 and 50 *μ*M [21]. The antiproliferative effect of quercetin is believed to be exerted by producing cell cycle arrest in G1 phase [22]. In our study, we have shown that quercetin inhibited papillary thyroid cancer cell proliferation and triggered apoptosis at 50 and 75 *μ*M concentrations, but cells were arrested in the S phase only at 75 *μ*M concentration.

Quercetin was found to have a differential effect on the cell cycle in different myeloid and lymphoid cell lines in the above-mentioned study [22]. Although their cell cycle data showed mainly G0/G1 arrest, some treatments arrested cells in S-phase and G2M. The mechanism of antiproliferative effect of quercetin in mammary and epidermoid cancer cells was investigated in a recent study by D'Archivio et al. [23]. These authors observed a biphasic effect similar to our findings and reported a dose dependent S phase arrest at higher concentrations of quercetin.

Apoptosis is a morphologically distinct form of programmed cell death. Impairments in apoptosis can be associated with several disease states including cancer. Thus, induction of apoptosis in cancer cells is a potentially promising approach for cancer therapy [4]. Several studies, including ours, have shown that quercetin and other flavonoids have therapeutically relevant properties such as induction of apoptosis in tumor cells as well as antiviral, antioxidant, anti-inflammatory, and antiproliferative activities [9–11, 17, 21, 24]. To determine relevancy of these* in vitro* effects of polyphenols to their clinical use, it is essential to consider their bioavailability and whether these relatively high concentrations are achievable in plasma. It has been suggested that physiological concentrations of polyphenols and their metabolites in plasma will not exceed 10 *μ*M [25, 26]. Data on bioavailability of polyphenols is still limited, but there is evidence that quercetin obtained from plant products can result in micromolar concentrations in blood plasma [27]. It is also remarkable to note that published data on quercetin pharmacokinetics in humans suggest that a dietary supplement of 1-2 g of quercetin may result in plasma concentrations between 10 and 50 *μ*M and that these concentrations induce apoptosis and downregulate antiapoptotic proteins* in vitro* [28]. Apoptosis can be triggered by caspases. Caspases, represented by a family of cysteine proteases, are the key proteins that modulate the apoptotic response. Among them, caspase-3 is a key protein of apoptotic mechanisms and the activated caspase-3 is responsible for the morphological hallmarks of apoptosis, including DNA fragmentation and membrane blebbing [29]. We have measured the cleavage of caspase-3 and PARP and observed that quercetin-induced cell death was accompanied by an increase in the activity of caspase-3 which stimulated the molecular cascade of apoptosis and DNA fragmentation. Heat shock proteins (Hsps) are extremely conserved and have different roles in principal cellular processes. Hsp90 is a molecular chaperone and protects the organism from stress-induced cell injury. It has been reported to have a regulatory role on certain steps of the apoptotic cascade [5]. In this study, we have demonstrated that the ability of quercetin to induce apoptosis in B-CPAP cells was associated with its Hsp90 inhibitory activity. This effect might represent a mechanism by which polyphenolic dietary plant compounds may exert their anticancer effects.

Unlike normal cells, cancer cells have increased proteasomal activity that is essential for their survival and uninhibited proliferation [30, 31]. Proteasome activity is regulated via different regulators including Hsps. Among others, Hsp90 is mainly responsible for keeping proteins in a functional folded state. It also plays a role in the proteasomal degradation process. In addition to all these important functions, Hsp90 prevents protein aggregation [32]. In recent years, different polyphenols such as apigenin, epigallocatechin gallate, quercetin, and myricetin have been reported to act as proteasome inhibitors that induce cell death in cancer cells [33]. It was also reported that various fruit and vegetable extracts, particularly grape extract, are capable of inhibiting the proteasome activity and that this inhibition is associated with tumor cell apoptosis [34, 35]. We have also observed that quercetin could significantly inhibit the proteasome activity in a concentration-dependent manner where chymotrypsin-like proteasomal activity was decreased at all concentrations of quercetin. Additionally, our data may provide a link between Hsp90 downregulation and proteasome inhibition through increased protein aggregation.

## 5. Conclusion

Although research in recent years has generated new targeted therapies in thyroid cancer, a gap remains in the treatment of recurrent papillary cancers not amenable to operative resection. We have shown that quercetin downregulates the expression of Hsp90 and decreases chymotrypsin-like proteasome activity and that these effects are related to inhibition of growth and caspase dependent apoptotic cell death in papillary thyroid cancer cells. It is hypothesized that these effects lead to certain stress conditions in cells which trigger the apoptotic cell death mechanism via caspase-3 activation and PARP cleavage. Thus, Hsp90 inhibition appears to be an important approach for thyroid cancer drug development. However, the molecular mechanism of the antiproliferative and apoptotic effects of Hsp90 remains to be determined.
